# Air Quality Response in China Linked to the 2019 Novel Coronavirus (COVID‐19) Lockdown

**DOI:** 10.1029/2020GL089252

**Published:** 2020-10-06

**Authors:** K. Miyazaki, K. Bowman, T. Sekiya, Z. Jiang, X. Chen, H. Eskes, M. Ru, Y. Zhang, D. Shindell

**Affiliations:** ^1^ Jet Propulsion Laboratory California Institute of Technology Pasadena CA USA; ^2^ Japan Agency for Marine‐Earth Science and Technology Yokohama Japan; ^3^ School of Earth and Space Sciences University of Science and Technology of China Hefei China; ^4^ Royal Netherlands Meteorological Institute (KNMI) De Bilt the Netherlands; ^5^ Nicholas School of the Environment Duke University Durham NC USA; ^6^ Porter School of the Environment and Earth Sciences Tel Aviv University Tel Aviv Israel

**Keywords:** COVID‐19, air quality, NO_2_, ozone, health impact

## Abstract

Efforts to stem the spread of COVID‐19 in China hinged on severe restrictions to human movement starting 23 January 2020 in Wuhan and subsequently to other provinces. Here, we quantify the ancillary impacts on air pollution and human health using inverse emissions estimates based on multiple satellite observations. We find that Chinese NOx emissions were reduced by 36% from early January to mid‐February, with more than 80% of reductions occurring after their respective lockdown in most provinces. The reduced precursor emissions increased surface ozone by up to 16 ppb over northern China but decreased PM2.5 by up to 23 μg m^−3^ nationwide. Changes in human exposure are associated with about 2,100 more ozone‐related and at least 60,000 fewer PM2.5‐related morbidity incidences, primarily from asthma cases, thereby augmenting efforts to reduce hospital admissions and alleviate negative impacts from potential delayed treatments.

## Key Points

- We quantified the ancillary impacts of the COVID‐19 lockdown on air pollution and human health using multiple satellite observations
- Rapid reductions in Chinese NOx and SO_2_ emissions increased surface ozone by 16 ppb over northern China but decreased PM2.5 nationwide
- These changes increased by about 60 ozone‐related but decreased by about 5,000 PM2.5‐related hospital admissions

## 1 Introduction

On 23 January 2020, 2 days before the Chinese New Year (CNY) celebration, the Chinese government imposed a “lockdown”in Hubei province, which severely limited transportation and overall economic activity (Chinazzi et al., 2020; Li et al., 2020) until 8 April 2020 when the lockdown was lifted in Wuhan. These restrictions were designed to “flatten the curve” of disease transmission and consequently alleviate strain on the health care system (Wang et al., 2020). However, these mitigation efforts also had ancillary impacts on emissions of air pollutants, which represent the fifth highest mortality risk factor globally and are associated with about 4.9 million deaths in 2017 (Health Effects Institute, 2019). Particulate matter at 2.5 micron (PM2.5) and ozone are the primary contributors to air pollution. Ozone is formed through secondary photochemical production from precursor constituents such as hydrocarbons and carbon monoxide in the presence of nitrogen oxides (NOx), whereas PM is a widespread air pollutant including solid and liquid particles. During the 21st century, China has become the epicenter of a dramatic redistribution of air pollutant emissions (Miyazaki et al., 2017; Zheng et al., 2018). Consequently, changes there could lead to substantial impacts on regional and potentially global air quality.

Satellite measurements, such as NO_2_ columns from the Ozone Mapping Instrument (OMI) and the TROPOspheric Monitoring Instrument (TROPOMI), can readily capture synoptic changes in pollutants, including rapid reductions during the Chinese lockdown (Bauwens et al., 2020; Liu et al., 2020). However, the inference of emissions from these measurements must account for variations in atmospheric transport, chemical environment, and meteorology. To that end, advanced chemical data assimilation systems incorporate these factors through ingestion of multiple chemical satellite and in situ observations into chemical transport models (CTMs) (Miyazaki et al., 2019; Miyazaki, Bowman, Yumimoto, et al., 2020; Qu et al., 2019). The‐state‐of‐the‐art data assimilation of multiconstituent observations has the potential to improve emission inversions by accounting for confounding factors in the relationship between emissions and concentrations, while reducing model observation mismatches arising from model errors unrelated to emissions (Miyazaki et al., 2017).

We estimate NOx and SO_2_ emissions across all Chinese provinces accounting for the effects of emission reductions from CNY and the timing of lockdowns for each province. Changes in ozone and PM 2.5 are computed both within and between provinces. Consequently, health impacts, which we compute based upon population and exposure‐response relationships (Liang et al., 2018), account for the effect of both local and nonlocal emissions changes. For these estimates, we use a multiconstituent satellite data assimilation system (Miyazaki, Bowman, Sekiya, et al., 2020), which simultaneously optimizes concentrations and emissions of various species while taking their complex chemical interactions into account.

## 2 Data and Methods

An extended calculation of the Tropospheric Chemistry Reanalysis Version 2 (TCR‐2) (Miyazaki, Bowman, Sekiya, et al., 2020) is used to evaluate emission and concentration changes (Texts S1 and S2 in the supporting information). The data products used in this study have been obtained from the assimilation of multiple satellite measurements of ozone, CO, NO_2_, HNO_3_, and SO_2_ from the OMI, TROPOMI, MLS, and MOPITT satellite instruments (Text S3). The forecast model used is MIROC‐CHASER (Text S4). An ensemble Kalman filter technique was used to optimizes both chemical concentrations of various species and emissions of several precursors. Surface measurements of NO_2_, O_3_, and PM2.5 concentration data from the national air quality monitoring stations (NAQMS) stations (Text S5) were used to evaluate the assimilation results. For short‐term health impacts, we estimated respiratory hospital admissions (HAs) and asthma‐related emergency room visits (ERVs) for short‐term ozone exposure, and children asthma symptom days, children bronchitis, respiratory HAs, and cardiovascular HAs for short‐term PM2.5 exposure (Text S6).

## 3 Results

### 3.1 Anthropogenic Emission Reductions

**Figure 1:**
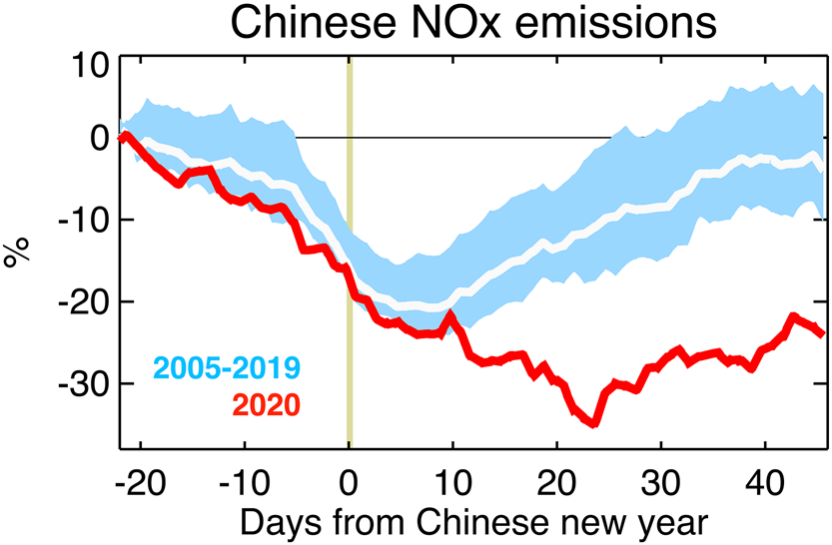
Time series of relative changes in Chinese NOx emissions (in %) derived using OMI measurements as a function of days from CNY. The results are shown for 2005–2019 (average by while line and 1‐*σ* standard deviation in light blue shade) and 2020 (red line).

Chinese emissions are typically low from January to February as a consequence of CNY. Climatological variations referenced to CNY in Figure 1 are derived from our 16‐year (2005–2020) emission time series (Miyazaki, Bowman, Sekiya, et al., 2020). These reductions start about 20 days beforehand and reach their nadir after CNY before recovering about a month later. This recovery is reflected both in NOx emissions (13% higher after the CNY holiday in the 2005–2019 average based upon a 14‐day average) and NO_2_ concentrations at the surface (+80.8% in 2019; Table S1). Consequently, the mean CNY emissions are about 1.4 TgN/year (daily emission values on a per year equivalent) less than the start of the year (9.0 TgN/year) in the 2005–2019 average. Over the last decade, there have been significant trends in emissions. From 2005 to 2011 there was a 30% increase followed by rapid decrease after 2013 as a consequence of emissions controls (Cui et al., 2016; Miyazaki et al., 2017). However, these trends do not impact the relative reductions from the start of the year (less than 5% multiyear spread).

In 2020, vehicle and industry activity were already affected by COVID‐19 before the holidays (Kraemer et al., 2020) consistent with observed emission reductions. Right after the Wuhan lockdown and during the national holiday for CNY (January 24 to February 2), emissions decreased by 0.9 to 6.2 TgN/year, which is about 26% lower than the value at the beginning of the year. The NOx emissions continued to decrease after the holidays and reached their minimum value of 5.5 TgN/year on 17 February, which is 36% smaller than the early January value. The peak emission reduction in 2020 (2.9 TgN/year) is about two times larger than that in the 2005–2019 average (1.4 TgN/year). The reduction in 2020 corresponds to about 9% of the global total anthropogenic emissions (33.4 TgN/year) on a daily basis, which is comparable to the total emissions from Europe (4.1 TgN/year), the United States (4.2 TgN/year), or India (3.4 TgN/year). Accounting for climatological variability, we attribute the additional 1.5 TgN/year reduction to COVID‐19 mitigation. By applying the average recovery rate per grid cell after 23 January (when the first lockdown was implemented), the accumulated emission amounts (total nitrogen emissions in NO_2_ released to the atmosphere) during February 2020 is reduced by about 16% using either the OMI assimilation (553 to 461 GgN) or the TROPOMI assimilation (378 to 316 GgN) using the same recovery rate linked to the lockdown. The relative emission changes derived using two instruments are consistent at country scale (Table S2).

**Figure 2:**
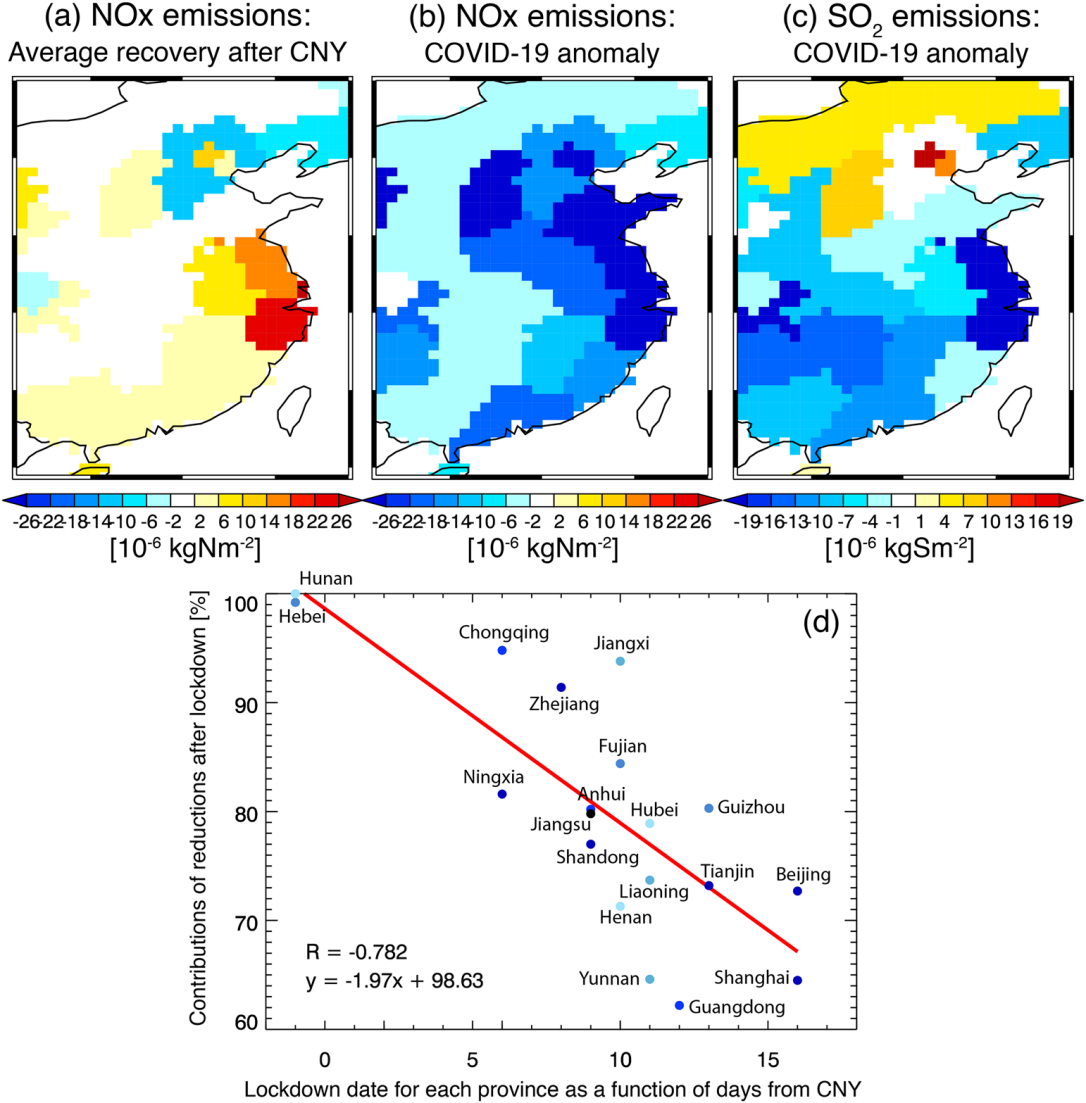
Spatial distributions of the NOx and SO_2_ total accumulated emission reductions from 23 January to 29 February 2020. The results are shown for (a) NOx emission changes due to average recovery rate for 2015–2019 and (b) due to COVID‐19 anomaly and (c) SO_2_ emission changes due to COVID‐19 anomaly. (d) Contributions of emission reductions after lockdown to the total NOx emission reductions from 23 January to 29 February 2020 (in %) for each province as a function of days from CNY. The red line and numbers show linear regressions. Each dot represents each province, while the different colors represent accumulated emission reductions corresponding to the results in Figures 2a and 2b.

**Figure 3:**
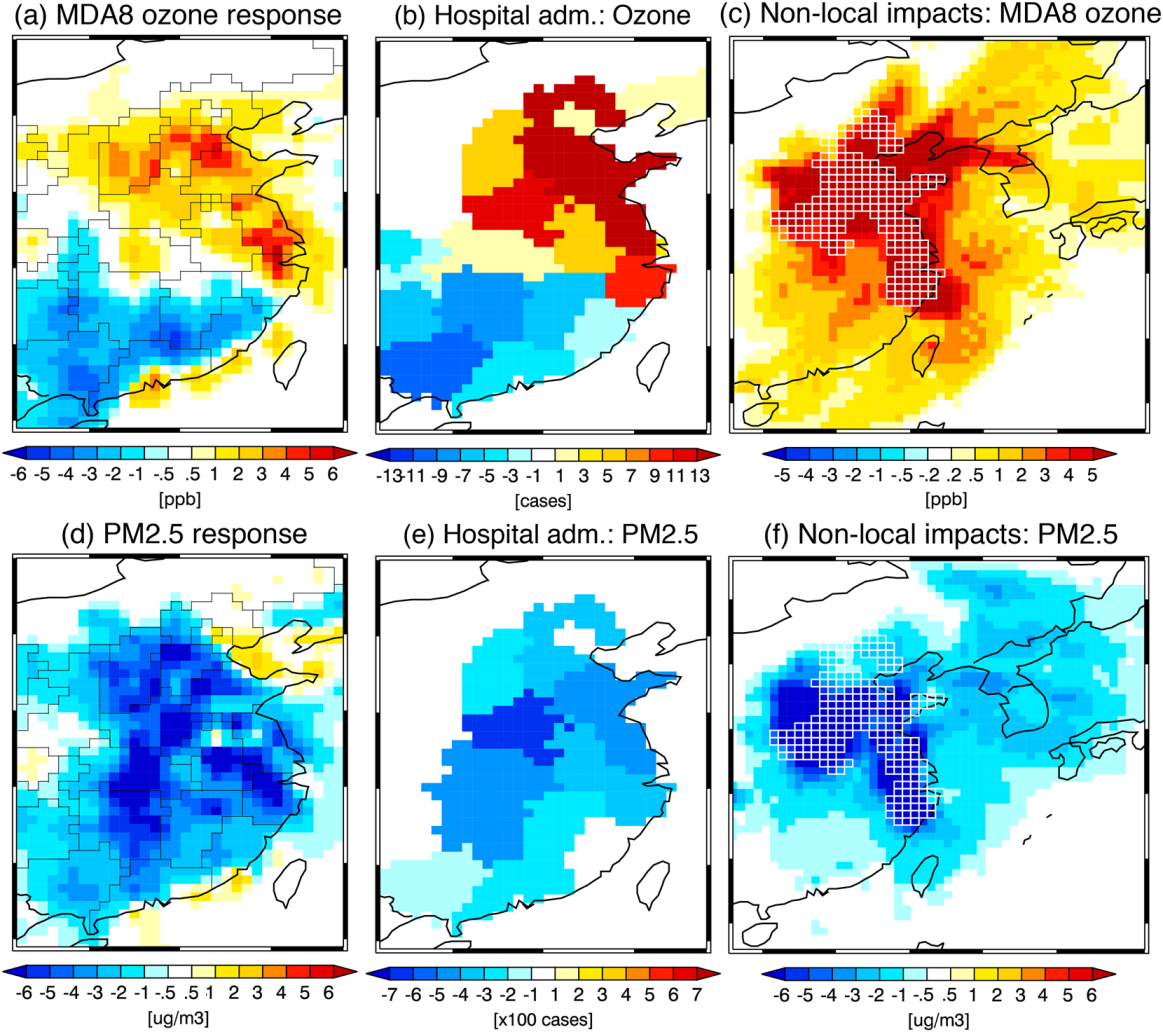
Changes in (a) MDA8 (in ppb) and (d) PM2.5 concentrations (in μg m^−3^) and (b, e) their impacts on short‐term exposure linked to the COVID‐19 lockdown during 15–25 February 2020. For (b) short‐term ozone exposure, respiratory HAs in post‐65 population are shown. For (e) short‐term PM2.5 exposure, the total number of respiratory and cardiovascular HAs is shown. The results are also shown for maximum concentration changes at each grid point during 15–25 February 2020 in (c) MDA8 (in ppb) and (f) PM2.5 (in μg m^−3^) linked to the lockdown for the five provinces in northeastern China (Zhejiang, Jiangsu, Shandong, Henan, and Hebei). The emission reductions were considered for the five provinces only in this case, which are marked by white mesh lines.

The baseline emission recovery for different provinces is shown in Figure 2a. We use the TROPOMI NO_2_ assimilation results for the spatial analysis because of its better spatial coverage than OMI, while using the average recovery rate from the OMI records (Figure 1). The recovery was on average about 3 × 10^−6^ kgN/m^2^ with some provinces such as Zhejiang exceeding 20 × 10^−6^ kgN/m^2^ after CNY. The impact of lockdown paints a very different picture. Rather than a recovery, provinces such as Zhejiang, Jiangsu, and Shandong along the eastern seaboard of China saw accumulated reductions exceeding 25 × 10^−6^ kgN/m^2^ from 23 January to 29 February due to the lockdown (Figure 2b). Spatially integrated country‐wide totals show a reversal from +9 GgN (area‐integrated emission sum of Figures 2a and 2b) to −57 GgN due to lockdown based upon the TROPOMI assimilation. Similarly, OMI assimilation shows a reduction from −4 to −100 GgN.

The NOx emission reductions are linked to the timing of the provincial lockdown. In the majority of provinces, 80% of reductions occurred after their respective lockdown (Figure 2d). For almost all provinces, 60% of reductions occurred after the lockdown. The relatively good linear relationship in Figure 3 of 2% per day reduction after the Wuhan lockdown (*r* = −0.78) suggest that the longer provinces waited to impose their own lockdown, the more impact neighboring provinces had on local emissions reductions. In addition, the highest level of emergency announcement was issued on 29 January to all Chinese provinces, which likely affected economic activity before the actual implementation of provincial lockdowns, which sustained the lower emission levels at least until 21 February when the emergency level was lifted.

The reduction in NO_2_ concentrations have significantly different spatial patterns than emissions (Table S3). The regional mean tropospheric NO_2_ columns from TROPOMI retrievals show a north‐south gradient with reductions of 50.6% and 38.2% for northeast and southeast China, respectively, from 4–14 January to 14–24 February, whereas the estimated emission reductions are more uniform at 35.0% and 37.1%. The differences between emission and concentration reductions underscore the importance of nonlinear chemistry (Miyazaki, Bowman, Yumimoto, et al., 2020). The north‐to‐south gradient in tropospheric NO_2_ reductions is largely different between OMI (33.9% and 42.0%) and TROPOMI (50.6% and 38.2%), highlighting the influences of sampling and retrieval errors, whereas the estimated emission changes are consistent for the two instruments and not largely affected by the retrieval differences.

Similar to NOx emissions, the estimated SO_2_ emissions exhibited a reduction and recovery pattern (Figure S1). The decreasing rate before the holiday is substantially larger in 2020 (−0.70 TgS/year) than the climatological average (−0.12 TgS/year). However, in contrast to a recovery in previous years (+0.32 TgS/year), SO_2_ emissions continue to decrease after the holiday (−0.24 TgS/year for 2 weeks after the holiday). The maximum SO_2_ emission reduction in January–February 2020 is 1.8 TgS/year (by 29%, from 6.2 to 4.4 TgS/year), which corresponds to about 5% of the global total emissions (33.0 TgS/year) and is comparable at an annual rate to the total emissions from India (1.8 TgS/year). In contrast to NOx emissions, SO_2_ emission reductions were concentrated in eastern and central China (Figure 2c), which could be attributed to different dominant emission categories. Power plant, industrial, and residential emissions dominate SO_2_ emissions (Zheng et al., 2018). The northern and southern contrast could reflect the continued use of residential coal in the northern part, whereas reductions in emissions from the power and industry sectors could lead to the reductions in the southern part. The increased SO_2_ emissions around the Beijing area will be further explored in future study.

### 3.2 Air Quality Changes and Short‐Term Health Impacts

In order to isolate the impact of lockdown on air pollutants, the 2020 emissions are adjusted based upon the difference between the 2015–2019 emission trends and 2020 emissions after CNY (Figure S3). Our results show a bifurcated response in daily maximum 8‐hr average (MDA8) ozone, which increased over central and northern China but decreased over southern China after the CNY holiday (Figure 3a). The MDA8 responses reached 6 ppb for the 15–25 February average and 16 ppb for a single day over Hebei on 19 and 20 February. In particular, the Jiangsu province near Shanghai and Shandong province south of Beijing showed elevated responses exceeding 5 ppb for the 15–25 February average. Conversely, southern China broadly had reductions in ozone by around 1–5 ppb with higher reductions in coastal provinces in near Hong Kong in spite of broadly comparable NOx emission reductions. The opposing responses can be explained in part by the removal of ozone through NOx titration, which is enhanced by less efficient NOx transport from the boundary layer and a slower rate of photochemical ozone production predominant in winter seasons. This phenomenon is largely responsible for ozone increases during the cold season in response to decreased NOx emissions along with VOCs changes (Jhun et al., 2014). The positive ozone response along the coastal Pearl River Delta region is also likely due to NOx titration (Yang et al., 2019).

These responses can differ significantly between CTMs. Those differences, however, can be diagnosed from our multimodel chemical data assimilation (Miyazaki, Bowman, Yumimoto, et al., 2020). The estimated ozone response (Text S7) had a factor of 2 difference among different models used within this framework due to fundamental differences in the representation of fast chemical and dynamical processes (Figure S4). For northern central China, the large negative responses range from 0.4–0.6 ppb per unit emission change (10^−11^ kgN m^−2^ s^−1^). Even with a range of models, the multimodel differences in MDA8 simulations are smaller than 6 ppb for most regions (Miyazaki, Bowman, Yumimoto, et al., 2020), which is smaller than the evaluated model bias against the in situ observations for most of eastern and southern China (Table S4). The biases suggest potential problems of many CTMs due to errors such as in dry deposition and VOCs emissions (Li et al., 2019). The uncertainty ranges in the Chinese NOx emissions due to model errors were quantified to be about 21% from the multimodel data assimilation, while showing consistent temporal variations.

Nevertheless, these responses are broadly consistent with observed surface ozone changes as summarized in Table S4 and Figure S2 and described in Text S8, which can be explained by the combination of emissions (Figure S3) and background variability (i.e., synoptic and seasonal changes). The observed large increase in northeastern China is strongly related to the emission reductions. For some parts of southern China, both the observed and simulated ozone started to increase before the CNY holiday (Figure S2) and continued afterward where the emission reductions do not solely explain the observed variability.

The PM2.5 response shows a strikingly different pattern than ozone (Figure 3d), with reductions of up to 10 μg m^−3^ for the 15–25 February average and up to 23 μg m^−3^ for a single day over Anhui on 20 February. Whereas the sign of the ozone response depends on region, the PM2.5 response to emissions decreases everywhere but is particular different in central China where provinces saw significant reductions such as Hubei (21 μg m^−3^) and Henan (30 μg m^−3^) (Table S4). In the model simulations, about 54%, 92%, and 71% of the reductions in sulfate, nitrate, and ammonium aerosol concentrations were associated with SO_2_ and NOx emission reductions after the 2020 CNY. However, model responses underestimated PM2.5 relative to surface sites, especially over northeastern China (Table S4). These underestimates could be explained by the lack of observational constraints on direct aerosol emissions of organic and black carbon and dust, and on other precursors such as NH_3_ and VOCs. Because aerosol secondary formations can be initiated by OH‐oxidation, changes in OH also affect the PM2.5 response. The surface OH concentrations were decreased by about 5–25% in southern China and increased by 10–50% in central and northern China, linked to the ozone and NOx changes (Figure S3). These are likely responsible for the relatively weak PM2.5 response in southern China, together with regional differences in removal processes, as also suggested by Huang et al. (2020) and Le et al. (2020). Aerosols have numerous impacts on surface ozone production and loss through heterogeneous chemical reactions, such as hydrolysis of N_2_O_5_, irreversible absorption of NO_2_ and NO_3_ on wet aerosols, and their influences on photolysis rate, which can reduce ozone by 8–20 ppb in northern and eastern China (Li et al., 2018, 2019; Lou et al., 2014). Thus, the underestimated PM2.5 variations, along with other factors such as VOCs, could have impacts on temporal changes in ozone. However, the impact of aerosols heterogeneous reactions is complex, while the model considered N_2_O_5_ hydrolysis and HO_2_ uptake but not NO_2_ and NO_3_ absorption on wet aerosols.

Reductions in ozone and PM2.5 from local emissions can impact pollutant distributions in other regions through atmospheric transport. We conducted a sensitivity calculation with changing emissions for five provinces in northern and eastern China (Zhejiang, Jiangsu, Shandong, Henan, and Hebei) where more than 70% of contributions to total emission reductions occurred after lockdown. Corresponding to these emission changes, MDA8 and PM2.5 outside the five provinces were increased by up to 7 ppb and decrease by up to 6 μg m^−3^, respectively, across East Asia including western Japan and Korean peninsula (Figures 3c and 3f). In southern China, the increases in ozone due to the nonlocal impacts compensated for parts of the ozone decreases due to the local emission reductions. For PM2.5, the nonlocal impacts lead to further reductions in southeastern China along with the local impacts.

The reductions in pollution from lockdown have a direct impact on human health, which are derived from population, baseline incidence rates for specific outcomes, and epidemiological exposure‐response functions. Given the short window of the studied period, we focused our health impacts assessment on short‐term effects associated with ozone and PM2.5. These short‐term effects were characterized as morbidity, that is, the disease, symptoms, and the required HAs and ERVs when necessary (Text S6), as shown in Figure 3, Tables 1 and S6 for HAs, and Table S5 for all cases. The short‐term exposure changes linked to the lockdown were estimated using the ozone and PM2.5 simulation results with the standard and modified emissions. For ozone, total asthma‐related HRVs for all ages and respiratory HAs in post‐65 population were estimated. For PM2.5, the number of respiratory and cardiovascular HAs for all ages and cases of bronchitis in children ages 6–12 and asthma symptom days in children ages 5–19 were estimated. The total increase of 2,105 (905–3,307, 95% confidence levels) incidence of HRVs and HAs due to ozone short‐term exposure during 15–25 February is attributed to strong increases in northeastern China with the most impacts in Shandong, Henan, and Hebei (Table S5). The changes in PM2.5, associated with reductions in secondary formation processes in our estimates, led to a decrease of 60,691 (37,897–83,503) incidences of PM2.5‐related asthma symptom days, cases of bronchitis, and HAs with the Top 3 contributions from Henan, Hunan, and Hebei (Table S5). In particular, changes in ozone and PM2.5 were associated with 5,017 reductions of respiratory and cardiovascular HAs, with the Top 3 contributions from Henan, Hunan, and Shandong (Table 1; Figure 3b and 3e). Values using a more limited set of China‐specific epidemiological results available for HAs related to PM exposure suggest responses near the low end of the ranges reported here (Text S6). Regardless of the choice of epidemiological results, the avoided HAs were of the same order of magnitude as those needed for COVID‐19 nationwide during the same period (∼2,165 based on an upper‐limit estimate; Text S9). In Hubei, where the majority of COVID‐19 hospitalization (∼2,019) occurred, the reduction of HAs due to air quality was estimated as 406, accounting for 20% of the upper limit of COVID‐19 HAs. This implies that the air pollution declines in response to the lockdown led to overall reductions in HAs due to air quality and alleviated negative impacts of potential delayed treatment. We caution that estimating HAs and ERVs during COVID‐19 period is complex as elective procedures were often suspended during the lockdown and people may have also preferred to avoid hospital visits. However, the implications on health impacts we showed above are likely to be robust, given the distinct scale of estimates. Given the direct emissions of aerosols were not explicitly constrained in our estimate with consequent underestimates relative to observed changes (Table S4), the actual impacts of PM2.5 changes are likely even larger.

**Table 1:** Total Values of Respiratory HA Changes for Short‐Term Ozone Exposure in Post‐65 Population (Ozone HA), Respiratory and Cardiovascular HAs for All Ages for Short‐Term PM2.5 Exposure (PM2.5 HA), and New COVID‐19 Cases for 15–25 February 2020

Province	Ozone HA	PM2.5 HA	COVID‐19 cases
Liaoning	1(0–3)	−46 (−108–18)	7
Beijing	3 (2–3)	−38 (−96–20)	25
Tianjin	2 (1–3)	−1 (−55–54)	15
Hebei	13 (10–17)	−371 (−605 to −137)	21
Shanxi	6 (5–8)	−223 (−315 to −132)	6
Shaanxi	1 (−1–3)	−46 (−136–43)	13
Shandong	17 (12–21)	−423 (−734 to −111)	226
Jiangsu	−5 (−8 to −3)	−243 (−331 to −156)	27
Shanghai	3 (2–4)	−38 (−81–5)	10
Anhui	6 (4–9)	−400 (−589 to −210 )	39
Henan	13 (8–18)	−696 (−1,032 to −359)	63
Hubei	3 (0–5)	−406 (−603 to −210)	10,976
Zhejiang	10 (7–13)	−338 (−442 to −235 )	43
Jiangxi	−5 (−8 to −3)	−243 (−331 to −156)	22
Hunan	−8 (−11 to −5)	−500 (−692 to −308)	15
Guizhou	−5 (−7 to −3)	−79 (−190 to 31)	3
Fujian	−3 (−5 to −1)	−78 (−115 to −41)	9
Guangdong	−4 (−9–0)	−265 (−407 to −123)	53
Guangxi	−11 (−13 to −9)	−155 (−230 to −80)	17
Total	60 (−4–124)	−5,077 (−8,640 to −1514)	11,769

*Note*. The 95% upper and lower confidence levels are also shown. HAs due to COVID‐19 can be estimated based on the COVID‐19 cases and the hospitalization fraction of COVID‐19 cases (∼2,165 cases for country total and ∼2,020 cases for Hubei based on an upper‐limit estimate). The results are shown for selected provinces and country total.

## 4 Conclusions and Discussion

The unprecedented steps taken to stop the transmission of COVID‐19 had the ancillary effect of rapid reductions in NOx and SO_2_ emissions, from 8.4 TgN/yr in early January to only 5.5 TgN/yr in mid‐February for NOx and from 6.2 to 4.4 TgS/year for SO_2_. These reductions provide insights but also challenges for future air quality policy and their interactions with chemistry‐climate projections, which could be assessed with approaches like hierarchical emergent constraints (Bowman et al., 2018). Our results show that emission reductions can have opposing responses on different air pollutants and that policies in one location can affect emissions in another (Figure 3). The bifurcated response in MDA8 ozone has commonly been shown using observations and models with a priori assumptions on emission activity and efficiency (Huang et al., 2020; Le et al., 2020; Shi & Brasseur, 2020; Zhao et al., 2020). Our observationally constrained emissions allowed us to represent detailed spatial and temporal evolution of emissions, concentrations, and subsequent health impacts in a consistent framework for the first time, without making any assumptions about emission changes. Other emission sources not constrained in this study, such as VOCs and carbonaceous aerosols, are likely affected by lockdown but vary differently than NOx and SO_2_ sources but are not as easily validated. Urban and rural chemistry along with point sources were not well separated here but could be improved in the future (de Foy et al., 2015; Valin et al., 2011).

Our results show that potential short‐term health impacts were significant with increases of about 2,100 ozone‐related but a decrease of about 60,000 PM2.5‐related incidences of morbidity, with a decrease of about 5,000 HAs. Henan was by far the greatest beneficiary of these reductions that was not directly reflected in the NO_2_ concentration reduction (Table 1). On the other hand, Shandong was the most negatively impacted from ozone exposure even though it did not have the largest ozone response. These health outcomes need to be placed in the context of this extraordinary event. For example, actual exposure given limited movement may be different as the balance of indoor and outdoor exposure would not be typical and as hospital visits during the lockdown were often limited by regulations and avoided by personal preference. These reductions were entirely a consequence of emissions‐related activity. Realizing similar improvements in emission efficiency would require significant changes in controls and technology. Nevertheless, the magnitude of estimated health impacts shows there are significant health benefits from such aggressive reductions in emissions that could serve as a basis for air quality planning in the future.

## Supporting information



Supporting Information S1

Data Set S1

Data Set S2

Data Set S3

## Data Availability

We acknowledge the use of data products from the NASA Aura and EOS Terra and Aqua satellite missions (from https://earthdata.nasa.gov at the following links: https://l0dup05.larc.nasa.gov/opendap/MOPITT/MOP02J.007/; https://acdisc.gesdisc.eosdis.nasa.gov/data/Aura_MLS_Level2/ML2HNO3.004/; https://acdisc.gesdisc.eosdis.nasa.gov/data/Aura_MLS_Level2/ML2O3.004/; and https://aura.gesdisc.eosdis.nasa.gov/data/Aura_OMI_Level2/OMSO2.003/). We also acknowledge the free use of the tropospheric NO_2_ column data from the SCIAMACHY, GOME‐2 and OMI sensors from https://www.qa4ecv.eu/ecv/no2‐pre and from TROPOMI. The data used in this paper are available for download (at https://ebcrpa.jamstec.go.jp/~miyazaki/data_GRL2020/).
