# Supplementary material for: Air Quality Response in China Linked to the 2019 Novel Coronavirus (COVID‐19) Lockdown
Source: Geophys Res Lett. 2020 Oct 6;47(19):e2020GL089252. doi: 10.1029/2020GL089252 (PMC7646019; doi:10.1029/2020GL089252)
Supplement: Supplementary file 1 — Supporting Information S1 [file GRL-47-e2020GL089252-s004.pdf]

Supporting Information for "Air quality response in China linked to the 2019 novel Coronavirus (COVID-19) lockdown"

K. Miyazaki[1], K. Bowman[1], T. Sekiya[2], Z. Jiang[3], X. Chen[3], H. Eskes[4], M. Ru[5], Y. Zhang[5], and D. Shindell[5,6]

[1]Jet Propulsion Laboratory, California Institute of Technology, Pasadena, CA, USA

[2]Japan Agency for Marine-Earth Science and Technology, Yokohama, 236-0001, Japan

[3]School of Earth and Space Sciences, University of Science and Technology of China, Hefei, Anhui, China

[4]Royal Netherlands Meteorological Institute (KNMI), De Bilt, the Netherlands

[5]Nicholas School of the Environment, Duke University, Durham, NC, USA

[6]Porter School of the Environment and Earth Sciences, Tel Aviv University, Israel

Contents of this file

1. Text S1 to S9
2. Figures S1 to S5
3. Tables S1 to S6

Additional Supporting Information (Files uploaded separately)

1. Captions for Datasets S1 to S3

Introduction

This supporting information provides descriptions of the data and methods used in this study.

Text S1. Chemical data assimilation

An extended calculation of the Tropospheric Chemistry Reanalysis version 2 (TCR-2) (Miyazaki et al., 2020a) is used to evaluate emission and concentration changes. The reanalysis products used in this study have been obtained from the assimilation of OMI and TROPOMI NO_2, MOPITT CO, OMI SO_2, and MLS ozone and HNO_3. The reanalysis calculation was conducted at 1.1° (for OMI assimilation) and 0.56° (for TROPOMI assimilation) horizontal resolution using a global chemical transport model MIROC-CHASER and an ensemble Kalman filter technique that optimizes both chemical concentrations of various species and emissions of several precursors. This approach was efficient for the correction of the entire tropospheric profile of various species and its year-to-year variations (Miyazaki et al., 2012, 2019, 2020a, 2020b). The quality of the reanalysis fields for 2005-2018 has been evaluated based on comparisons against independent aircraft, satellite, and ozonesonde observations for various chemical species on regional and global scales, as well as for seasonal, yearly, and decadal scales, from the surface to the lower stratosphere (Miyazaki et al., 2019, 2020b). The TCR-2 datasets are available at: https://doi.org/10.25966/9qgv-fe81.

Text S2. Emission estimation

The emission estimation is based on a state augmentation technique, which was employed in our previous studies (Miyazaki et al., 2012, 2017, 2019, 2020a, 2020b). In this

approach, the background error correlations, estimated from the ensemble model simulations, determine the relationship between the concentrations and emissions of related species for each grid point. This approach allows us to reflect temporal and geographical variations in transport and chemical reactions in the emission estimates. The state vector includes surface emissions of NOx, CO, and SO_2, lightning NOx sources, and the concentrations of various chemical species. The emissions in the state vector are represented by scaling factors for each surface grid cell at 0.56° (for TROPOMI assimilation) and 1.1 ° (for OMI assimilation) resolution. The a priori anthropogenic emissions of NOx, CO, and SO_2 were obtained from the HTAP version 2 for 2010 (Janssens-Maenhout1 et al., 2015), which were produced using the Regional Emission Inventory in Asia (REAS) for China. Emissions from biomass burning were based on the monthly Global Fire Emissions Database (GFED) version 4 (Randerson et al., 2018) for NOx and CO. Emissions from soils were based on monthly mean of the Global Emissions Inventory Activity (GEIA) (Graedel et al., 1993) for NOx. For other compounds, emissions were taken from the HTAP version 2 and GFED version 4 emissions.

Text S3. Satellite observations

The tropospheric NO_2 column retrievals from the QA4ECV version 1.1 level 2 product for OMI (Boersma et al., 2018) and TM5-MP-DOMINO version 1.2 for TROPOMI (van Geffen et al., 2020) were used to constrain NOx emissions. The ground pixel sizes are 13km×24km for OMI and 7km×3.5km for TROPOMI with local overpass times of 13:30 for both satellites. Low-quality data were excluded by applying the provided quality flag and information on solar zenith angle ($> 80°$), cloud radiance fraction (< 0.5), and air

mass factor (tropospheric air mass factor/geometric air mass factor$>$ 0.2). We employed a super-observation approach (Miyazaki et al., 2012) to generate representative data with a horizontal resolution of the forecast model (0.56° for TROPOMI and 1.1° for OMI). The OMI SO_2 data used were the planetary boundary layer vertical column SO_2 L2 product obtained with the principal component analysis algorithm (Krotkov et al., 2016). Only clear-sky OMI SO_2 data (cloud radiance fraction$<$ 20%) with solar zenith angles less than 70° were used, and the first and last ten cross-track positions were excluded to limit the across-track pixels. The MOPITT total column CO data used were the version 7 L2 TIR/NIR product (Deeter et al., 2017). The version 4.2 ozone and HNO_3 L2 products from MLS (Livesey et al., 2018) were used to constrain the chemical concentrations in the upper troposphere and lower troposphere, which were important in correcting the influences of stratospheric intrusions on tropospheric ozone.

Text S4. Chemical transport model, MIROC-CHASER

The forecast model used is MIROC-Chem (Sudo et al., 2002, Watanabe et al., 2011, Sekiya et al., 2018). It simulates spatial and temporal variations in chemical species in the troposphere and stratosphere, by calculating tracer transport (advection, cumulus convection, and vertical diffusion), emissions, dry and wet deposition, and chemical processes (92 species, 262 reactions) including the ozone-HOx-NOx-CH4-CO system with non-methane volatile organic compounds oxidation. It also includes stratospheric chemistry such as halogen chemistry. The meteorological fields were calculated using the MIROC-AGCM atmospheric general circulation model (Watanabe et al., 2011). To reproduce past me-

teorological fields and calculate short-term variability, the simulated meteorological fields were nudged to the 6-hourly ERA-Interim reanalysis data (Dee et al., 2011).

Text S5. NAQMS surface measurements

We collected surface-level NO_2, O_3, and PM2.5 concentration data from the national air quality monitoring stations (NAQMS) stations (http://106.37.208.233:20035/). These real-time monitoring stations have the ability to report hourly concentrations of criteria pollutants from over 360 cities. The daily data were averaged to obtain mean concentrations for each period. Following the previous study (Jiang et al., 2018), the random and sampling error in the mean concentrations is calculated using the bootstrapping method (Efron, 1979), which is a statistical re-sampling method for uncertainty estimations. Firstly, a bootstrap sample is generated by randomly drawing N data points from the full set of N data points. For example, the NAQMS NO_2 data was firstly averaged for each period, which produces a dataset with 335 stations (East China); N = 335 data points were then randomly drawn from the dataset; Average of NAQMS NO_2 over the East China is calculated as the mean value of the randomly drawn N = 335 data points. Secondly, the above process is repeated for various periods to produce the averages of NAQMS NO_2 over East China. These steps were repeated by 1000 times. Because the averages are based on the randomly drawn data points, we have different averages for the same period. The standard deviation of the ensemble of averages represent the uncertainty in the mean NAQMS NO_2 concentrations.

Text S6. Health impact assessment

We used an established approach to estimate short-term effects of ozone and PM2.5.

Δ Health impacts= $y_0 \times (1 - exp^{-\beta \Delta Exposure}) \times Population$

where health impacts include respiratory hospital admissions (HAs) and asthma-related emergency room visits (ERVs) for short-term ozone exposure, and children asthma symptom days, children bronchitis, respiratory HAs, and cardiovascular HAs for short-term PM2.5 exposure. y_0 is the baseline rates of each health impact mentioned above in China; β is the exposure-response function for unit change of exposure; population is the number of people in the susceptible age-group living in the same $0.5° \times 0.5°$ grid box with exposure estimates. For asthma ERV, y_0 is the baseline asthma-related ERVs, which is calculated as the product of baseline asthma prevalence and the fraction of ERVs among asthma patients (Anenberg et al., 2018).

Exposure-response functions are derived from existing studies. We first examined studies based in China. We found several cohort and time-series studies (Lu et al., 2020, Tian et al., 2018, 2019a, 2019b) conducted in China that estimated the health effects of PM2.5 on outpatient asthma visits, HAs for all cause and cause-specific cardiovascular disease (including one study specifically on ischemic stroke), and all-cause HAs. Notably, some of these studies indicate a relatively lower effect size compared to epidemiological studies from Europe and North America. However, these studies cover only a subset of known morbidity effects. Globally, morbidity effects such as asthma symptom days and bronchitis for children related to PM2.5, and asthma ERVs and respiratory HAs related to ozone have also been evaluated in large scale health impact assessments and are included in the analysis presented here. As a result, we develop two sets of morbidity estimates: the "China-specific" estimates based on evidence from China, and the "gen-

eral" estimates based on global scale multi-national results. The "China-specific" case may more accurately reflect the health effects in China, while the "general" case covers more health endpoints and is also better established as it relies upon meta-analyses of multiple epidemiological studies.

We developed the "China-specific" estimates based on China-based epidemiological studies. In addition to the aforementioned individual studies, we found a systematic review focused on effects of PM2.5 related to hospital utilization in China (Lu et al. 2015). It concluded that the China-specific effects can be close to the lower-end of estimates for European countries and the United States, even though overall magnitudes of effects are comparable to the global estimates. As such, we used a relative risk (RR) value 1.0026 (1.0017, 1.0035) based on Tian et al. (2019b) for all-cause cardiovascular HAs. We did not calculate the cause-specific cardiovascular HAs (such as ischemic stroke as presented in Tian et al. (2018)), because our goal is to assess the total burdens. For asthma, we calculated both HAs and asthmatic symptom days to address the total burdens that include different levels of severity. However, we thus did not include outpatient visits for asthma (as presented in Lu et al. (2020)), because of the likely overlap with HAs and asthmatic symptom days.

In addition, we develop our "general" estimates based on established global exposure-response functions. Priority is given to studies that are either systematic reviews of multiple studies, or large-scale multi-country studies. For respiratory HAs, we used RR of 1.006 (95% CI: 1.0008, 1.012) per 10 ppb derived from Air Pollution and Health: a European and North American Approach (APHENA) (Katsouyanni et al., 2009) on post-

65 population. Although this function was not directly observed from China, APHENA covered 149 countries in Europe and the US, while indicating no distinguishable geographical differences. For asthma ERVs, we used the averaged values of RR from three systematic reviews (1.03 (1.01, 1.06) (Orellano et al., 2017); 1.05 (1.04, 1.07) (Zhang et al., 2016); 1.02 (1.01, 1.02) (Zheng et al., 2015), all for per 10 ppb). For short-term effects of PM2.5, we used response functions from Carbon Reduction Benefits on Health (CaRBonH) calculation tool (Spadaro et al., 2018), which were originally developed for 53 countries in Europe and central Asia. CaRBonH systematically studied seven types of morbidity effects. We excluded adult chronic bronchitis due to the long-term nature of its development, and excluded restrictive activity days and work loss days, due to the essential shutdown of normal work and activities during the COVID-19 pandemic.

Baseline rates of respiratory and cardiovascular hospitalization in China was extracted from China health and family planning statistics yearbook 2016 (NHFPC, 2016). Baseline prevalence rate of asthma and bronchitis by age-group were from GBD 2018 database (Global Burden of Disease Collaborative Network, 2018). Population data by age-groups was obtained from GPW version 4 (Center for International Earth Science Information Network, 2018). The 95% upper and lower confidence levels were estimated for the selected provinces and country-total values (Table 1, S5, and S6) using the provided RR and baseline rate ranges.

Because the China-specific information is still very limited for our exposure estimates as discussed above, we present the "general" estimates based on established global exposure-response functions in the main text and SI (Table 1 and S5), while also discussing the

"China-specific" case results for the PM2.5 cardiovascular HAs in the SI (Table S5). The comparison between the "China-specific" and "general" results demonstrates that the China-specific values are close to the lower-end of the global estimates for the PM2.5 cardiovascular HAs (Table S5). Impacts of using China-specific values for other endpoints need to be further explored as comparisons between China-specific values and those from studies elsewhere do not show a systematic difference (Burnett et al., 2018).

Before the health impact assessments, a time-constant bias correction was applied to MDA8 based on the validation against the in-situ observations (Table S4), while the observed temporal changes were already broadly reproduced by the model for MDA8. As summarized in Table S6, the time-constant concentration bias correction had only slight impacts on the respiratory HA changes. We also evaluated the impact of uncertainty in the estimated concentration bias by applying random numbers (n=1000) to the standard deviation of model bias within each province using daily concentrations based on the Monte Carlo approach. The uncertainty in the exposure-response functions had larger impacts than those in the model concentration bias uncertainty. We also tested a time-constant bias correction for PM2.5, and obtained larger impacts in the exposure-response function uncertainty for both ozone and PM2.5-related HA estimates. As discussed in the main text, the underestimated temporal changes in the model PM2.5 concentration is likely due to the lack of observational constraints on direct aerosol emissions. Because we attempted to demonstrate the impacts of NOx and SO2 emission changes on human health through the secondary formation processes, a temporally-varying bias correction

was not tested, which would provide larger impacts on the estimated exposure changes than the time-constant bias correction.

As noted in the main text, hospital visits during the lockdown were often limited by regulations and avoided by personal preference, hence the results should be seen an indication of the extent of health impacts due to changing exposures to ozone and PM2.5, instead of the actual numbers of visits.

Text S7. Ozone production efficiency estimates using multi-model multi-constituent chemical data assimilation

Data assimilation that relies on a single model may lead to biased estimation of emissions and model response. We used the multi-model multi-constituent chemical data assimilation (MOMO-Chem) framework (Miyazaki et al., 2020b) to estimate response of surface ozone concentration to NOx emissions and its uncertainty. This system integrates a portfolio of data assimilation analyses obtained using four forward CTMs (GEOS-Chem, AGCM-CHASER, MIROC-Chem, MIROC-Chem-H) in a state-of-the-art ensemble Kalman filter data assimilation system. The framework was used to demonstrate the importance of the performance of forecast models for tropospheric chemistry data assimilation and to provide multi-model integrated information on the tropospheric chemistry system. By applying linear regressions to the ozone increments with respect to the NOx emission analysis increments using the daily mean data assimilation outputs at each grid point from MOMO-Chem, model responses of surface ozone to NOx emissions was evaluated (Miyazaki et al., 2020b). We first produced the daily multi-model integrated fields at each grid point and then applied them to linear regressions.

Text S8. Model and observation comparisons

Data assimilation improved agreements with the assimilated satellite NO_2 retrievals (Fig S5), with large reductions in model positive biases over polluted areas. In the OMI (TROPOMI) assimilation, the regional mean bias compared to the assimilated measurements are reduced by 91-94% (85-99%) by data assimilation, while root-mean-square-error (RMSE) are reduced by 68-93% (78-90%). The agreements against the assimilated measurements confirm that the observational constraints were sufficient to reproduce the observed variability through emission optimization. The linear regression slope against the NAQMS surface NO_2 measurements was 0.88-0.95 (0.41-0.50) for OMI (TROPOMI) assimilation over northeastern and southeastern China. The TROPOMI standard product shows negative biases against the OMI product (Ialongo et al., 2020).

Text S9. Hospital admissions (HAs) due to COVID-19

We evaluated HAs due to COVID-19 during the analysis period (February 15-25, 2020) based on COVID-19 cases and the hospitalization fraction of COVID-19 cases. The total reported number of COVID-19 new cases for the analysis period is 11,769 with 10,976 cases in Hubei (Table 1). The proportion of all infections that would lead to hospitalisation is estimated at 1.04 % for 20-20 years, 3.43 % for 30-39 years, 4.25 % for 40-49 years, 8.16 % for 50-59 years, 11.8 % for 60-69 years, 16.6 % for 70-79 years, and 18.4 % over 80 years (Verity et al., 2020). By applying a fraction of 18.4 %, an upper limit of HAs due to COVID-19 for the analysis period is estimated at 2,165 for country-total and 2,019 for Hubei.

The temporal changes in ozone during the analysis period are overall consistent between the data assimilation analysis and in-situ observation at the province scale (Table S4), with increases of 5-14 (3-14) ppb over northeastern China in the observation (model) and slight decreases over southern China in both data. The model bias only slightly varied with time for most regions. The temporal changes in model bias (i.e., model bias standard deviation) are much smaller than those of the observed concentrations at the provincial scale (Table S4). For individual stations, the model tends to underestimate variability, likely due to the representation error, with the linear regression slope of 0.4-0.47 and coefficient of 0.38-0.47 for the strong emission areas such as Jiangsu, Hunan, Jiangxi, Hubei, and Shandong. The general model performance for PM2.5 and aerosol concentrations have also been evaluated against in-situ observations and compared with other models (Bian et al., 2017; Liang et al., 2018).

References

Anenberg, S. C., D. K. Henze, V. Tinney, P. L. Kinney, W. Raich, N. Fann, C. S. Malley, H. Roman, L. Lamsal, B. Duncan, R. V. Martin, A. van Donkelaar, M.Brauer, R. Doherty, J.E. Jonson, Y. Davila, K. Sudo, J.C. Kuylenstierna, Estimates of the Global Burden of Ambient, Ozone, and on Asthma Incidence and Emergency Room Visits. *Environ Health Perspect.*, **126**, 107004 (2018)

Boersma, K. F., H. J. Eskes, A. Richter, I. De Smedt, A. Lorente, S. Beirle, J. H. G. M. van Geffen, M. Zara, E. Peters, M. Van Roozendael, T. Wagner, J. D. Maasakkers, R. J. van der A, J. Nightingale, A. De Rudder, H. Irie, G. Pinardi, J.-C. Lambert, S. C. Compernolle, Improving algorithms and uncertainty esti-

mates for satellite NO_2 retrievals: results from the quality assurance for the essential climate variables (QA4ECV) project. *Atmos. Meas. Tech.*, **11**, 6651–6678, https://doi.org/10.5194/amt-11-6651-2018 (2018)

Burnett, R., Chen, H., Szyszkowicz, M., Fann, N., Hubbell, B., Pope, C.A., Apte, J.S., Brauer, M., Cohen, A., Weichenthal, S., Coggins, J., Di, Q., Brunekreef, B., Frostad, J., Lim, S.S., Kan, H., Walker, K.D., Thurston, G.D., Hayes, R.B., Lim, C.C., Turner, M.C., Jerrett, M., Krewski, D., Gapstur, S.M., Diver, W.R., Ostro, B., Goldberg, D., Crouse, D.L., Martin, R.V., Peters, P., Pinault, L., Tjepkema, M., van Donkelaar, A., Villeneuve, P.J., Miller, A.B., Yin, P., Zhou, M., Wang, L., Janssen, N.A.H., Marra, M., Atkinson, R.W., Tsang, H., Quoc Thach, T., Cannon, J.B., Allen, R.T., Hart, J.E., Laden, F., Cesaroni, G., Forastiere, F., Weinmayr, G., Jaensch, A., Nagel, G., Concin, H., Spadaro, J.V., Global estimates of mortality associated with long-term exposure to outdoor fine particulate matter. *Proc. Natl. Acad. Sci.*, **115**, 9592-9597 (2018)

Center for International Earth Science Information Network - CIESIN - Columbia University, *Gridded Population of the World, Version 4 (GPWv4): Population Count, Revision 11* (NASA Socioeconomic Data and Applications Center (SEDAC), https://doi.org/10.7927/H4JW8BX5 (2018)

Dee, D. P., S. M. Uppala, A. J. Simmons, P. Berrisford, P. Poli, S. Kobayashi, U. Andrae, M. A. Balmaseda, G. Balsamo, P. Bauer, P. Bechtold, A. C. M. Beljaars, L. van de Berg, J. Bidlot, N. Bormann, C. Delsol, R. Dragani, M. Fuentes, A. J. Geer, L. Haimberger, S. B. Healy, H. Hersbach, E. V. Hólm, L. Isaksen, P. Kållberg, M.

Köhler, M. Matricardi, A. P. McNally, B. M. Monge-Sanz, J.-J. Morcrette, B.-K. Park, C. Peubey, P. de Rosnay, C. Tavolato, J.-N. Thépaut, F. Vitart, The ERA-Interim reanalysis: configuration and performance of the data assimilation system, *Q. J. R. Meteorol. Soc.*, **137**, 553–597, https://doi.org/10.1002/qj.828 (2011)

Deeter, M. N., D. P. Edwards, G. L. Francis, J. C. Gille, S. Martínez-Alonso, H. M. Worden, C. Sweeney, A climate-scale satellite record for carbon monoxide: the MOPITT Version 7 product. *Atmos. Meas. Tech.*, **10**, 2533–2555, https://doi.org/10.5194/amt102533-2017 (2017)

Efron, B., Bootstrap Methods - Another Look at the Jackknife. *Annals of Statistics*, **7**, 1–26 (1979)

Global Burden of Disease Collaborative Network. Global Burden of Disease Study 2017 (GBD 2017) Results. (Seattle, United States: Institute for Health Metrics and Evaluation (IHME) (2018)

Graedel, T. E., T. S. Bates, A. F. Bouwman, D. Cunnold, J. Dignon, I. Fung, D. J. Jacob, B. K. Lamb, J. A. Logan, G. Marland, P. Middleton, J. M. Pacyna, M. Placet, C. Veldt, A compilation of inventories of emissions to the atmosphere. *Global Biogeochem. Cy.*, **7**, 1–26 (1993)

Ialongo, I., Virta, H., Eskes, H., Hovila, J., and Douros, J., Comparison of TROPOMI/Sentinel-5 Precursor NO2 observations with ground-based measurements in Helsinki, *Atmos. Meas. Tech.*, **13**, 205–218, https://doi.org/10.5194/amt-13-205-2020 (2020)

Janssens-Maenhout1, G., M. Crippa1, D. Guizzardi, F. Dentener, M. Muntean, G. Pouliot, T. Keating, Q. Zhang, J. Kurokawa, R. Wankmüller, H. Denier van der Gon, J. J. P. Kuenen, Z. Klimont, G. Frost, S. Darras, B. Koffi, and M. Li, HTAP v2.2: a mosaic of regional and global emission grid maps for 2008 and 2010 to study hemispheric transport of air pollution. *Atmos. Chem. Phys.*, **15**, 11411–11432, doi:10.5194/acp-15-11411-2015 (2015)

Jiang, Z., B. C. McDonald, H. Worden, J. R. Worden, K. Miyazaki, Z. Qu, D. K. Henze, D. B. A. Jones, A. F. Arellano, E. V. Fischer, L. Zhu, K. F. Boersma, Unexpected slowdown of US pollutant emission reduction in the past decade. *Proc. National. Acad. Sci.* **115**, 201801191 (2018)

Krotkov, N. A., C. A. McLinden, C. Li, L. N. Lamsal, E. A. Celarier, S. V. Marchenko, W. H. Swartz, E. J. Bucsela, J. Joiner, B. N. Duncan, K. F. Boersma, J. P. Veefkind, P. F. Levelt, V. E. Fioletov, R. R. Dickerson, H. He, Z. Lu, D. G. Streets, Aura OMI observations of regional SO2 and NO_2 pollution changes from 2005 to 2015. *Atmos. Chem. Phys.*, **16**, 4605–4629, `https://doi.org/10.5194/acp-1646052016` (2016)

Liang, C.-K., J. J. West, R. A. Silva, H. Bian, M. Chin, Y. Davila, F. J. Dentener, L. Emmons, J. Flemming, G. Folberth, D. Henze, U. Im, J. E. Jonson, T. J Keating., T. Kucsera, A. Lenzen, M. Lin, M. T. Lund, X. Pan, R. J. Park, R. B. Pierce, T. Sekiya, K. Sudo, T. Takemura, HTAP2 multi-model estimates of premature human mortality due to intercontinental transport of air pollution and emission sectors. *Atmos. Chem. Phys.*, **18**, 10497–10520, `https://doi.org/10.5194/acp-18-10497-2018` (2018)

Livesey, N., W. Read, P. Wagner, L. Froidevaux, A. Lambert, G. Manney, L. Millán Valle, H. Pumphrey, M. Santee, M. Schwartz, S. Wang, R. A. Fuller, R. F. Jarnot, B. W. Knosp, E. Martinez, R. R. Lay, *Version 4.2 x Level 2 data quality and description document.* Jet Propul, Tech. rep., Lab., Tech. Rep. JPL D-33509 Rev. D, Pasadena, CA, USA (Available from `https://mls.jpl.nasa.gov/data/v4-2_data_quality_document.pdf`) (2018)

Lu, F., Xu, D., Cheng, Y., Dong, S., Guo, C., Jiang, X. and Zheng, X., Systematic review and meta-analysis of the adverse health effects of ambient PM2. 5 and PM10 pollution in the Chinese population. *Environmental research*, **18**, 196-204, (2015)

Lu, P., Zhang, Y., Lin, J., Xia, G., Zhang, W., Knibbs, L.D., et al., Multi-city study on air pollution and hospital outpatient visits for asthma in China. *Environmental Pollution*, **257**,113638 (2020)

Miyazaki, K., H. Eskes, K. Sudo, K. F. Boersma, K. Bowman, Y. Kanaya, Decadal changes in global surface NOx emissions from multi-constituent satellite data assimilation. *Atmos. Chem. Phys.* **17**, 807–837, `https://doi.org/10.5194/acp-17-807-2017` (2017)

Miyazaki, K., K. W. Bowman, K. Yumimoto, T. Walker, K. Sudo, Evaluation of a multi-model, multi-constituent assimilation framework for tropospheric chemical reanalysis. *Atmos. Chem. Phys.*, **20**, 931–967, `https://doi.org/10.5194/acp-20-931-2020` (2020b)

Miyazaki, K., Sekiya, T., Fu, D., Bowman, K. W., Kulawik, S. S., Sudo, K., Walker, T., Kanaya, Y., Takigawa, M., Ogochi, K., Eskes, H., Boersma, K. F., Thompson, A.

M., Gaubert, B., Barre, J., Emmons, L. K., Balance of emission and dynamical controls on ozone during KORUS-AQ from multi-constituent satellite data assimilation. *J. Geophys. Res.-Atmos.*, **124**, 387–413, https://doi.org/10.1029/2018JD028912 (2019)

Miyazaki, K., K. Bowman, T. Sekiya, H. Eskes, F. Boersma, H. Worden, N. Livesey, V. H. Payne, K. Sudo, Y. Kanaya, M. Takigawa, K. Ogochi, An updated tropospheric chemistry reanalysis and emission estimates, TCR-2, for 2005–2018. *Earth Syst. Sci. Data Discuss.*, https://doi.org/10.5194/essd-2020-30, in review (2020a)

Miyazaki, K., H. J. Eskes, K. Sudo, Global NOx emission estimates derived from an assimilation of OMI tropospheric NO_2 columns. *Atmos. Chem. Phys.*, **12**, 2263–2288, https://doi.org/10.5194/acp-12-2263-2012 (2012)

NHFPC, China health and family planning statistical yearbook (Beijing, China, Peking Union Medical College Publishing House, 2016)

Orellano, P., N. Quaranta, J. Reynoso, B. Balbi, J. Vasquez, Effect of outdoor air pollution on asthma exacerbations in children and adults: Systematic review and multilevel meta-analysis. *PLoS One*, **12**, e0174050 (2017)

Randerson, J. T., G. R. van der Werf, L. Giglio, G. J. Collatz, P. S. Kasibhatla, Global Fire Emissions Database, Version 4, (GFEDv4). ORNL DAAC, Oak Ridge, Tennessee, USA, https://doi.org/10.3334/ORNLDAAC/1293 (2018)

Spadaro, J. V., V. Kendrovski, G.S. Martinez, Achieving health benefits from carbon reductions: Manual for CaRBonH calculation tool. World Health Organization (2018)

Sekiya, T., K. Miyazaki, K. Ogochi, K. Sudo, M. Takigawa, Global high-resolution simulations of tropospheric nitrogen dioxide using CHASER V4.0, *Geoscientific Model Development*, **11**, 959–988, https://doi.org/10.5194/gmd-11-959-2018 (2018)

Shi, X., and Brasseur, G. P., The response in air quality to the reduction of Chinese economic activities during the COVID-19 outbreak. *Geophysical Research Letters*, 47, e2020GL088070. https://doi.org/10.1029/2020GL088070 (2020)

Sudo, K., M. Takahashi, J. Kurokawa, H. Akimoto, CHASER: A global chemical model of the troposphere 1. Model description, *J. Geophys. Res.*, **107**, ACH 7–1–ACH 7–20, https://doi.org/10.1029/2001JD001113 (2002)

Tian, Y., Liu, H., Zhao, Z., Xiang, X., Li, M., Juan, J., et al., Association between ambient air pollution and daily hospital admissions for ischemic stroke: A nationwide time-series analysis. *PLOS Medicine*, **15**: e1002668 (2018)

Tian, Y., Liu, H., Liang, T., Xiang, X., Li, M., Juan, J., et al., Fine particulate air pollution and adult hospital admissions in 200 Chinese cities: a time-series analysis. *International Journal of Epidemiology*, **48**:1142-1151 (2019a)

Tian, Y., Liu, H., Wu, Y., Si, Y., Song, J., Cao, Y., et al., Association between ambient fine particulate pollution and hospital admissions for cause specific cardiovascular disease: time series study in 184 major Chinese cities. *BMJ*, **367**:l6572 (2019b)

van Geffen, J., K. F. Boersma, H. Eskes, M. Sneep, M. ter Linden, M. Zara, J. P. Veefkind, S5P TROPOMI NO_2 slant column retrieval: method, stability, uncertainties and comparisons with OMI. *Atmos. Meas. Tech.*, **13**, 1315–1335, https://doi.org/10.5194/amt-13-1315-2020 (2020)

Verity, R., L. C Okell, I. Dorigatti, P. Winskill, C. Whittaker, N. Imai, G. Cuomo-Dannenburg, H. Thompson, P. G. T. Walker, H. Fu, A. Dighe, J. T Griffin, M. Baguelin, S. Bhatia, A. Boonyasiri, A. Cori, Z. Cucunubá, R. FitzJohn, K. Gaythorpe, W. Green, A. Hamlet, W. Hinsley, D. Laydon, G. Nedjati-Gilani, S. Riley, S. van Elsland, E. Volz, H. Wang, Y. Wang, X. Xi, C. A Donnelly, A. C Ghani, N. M Ferguson, Estimates of the severity of coronavirus disease 2019: a model-based analysis, *The Lancet* Published online March 30, 2020, https://doi.org/10.1016/S1473-3099(20)30243-7 (2020)

Watanabe, S., T. Hajima, K. Sudo, T. Nagashima, T. Takemura, H. Okajima, T. Nozawa, H. Kawase, M. Abe, T. Yokohata, T. Ise, H. Sato, E. Kato, K. Takata, S. Emori, M. Kawamiya, *MIROC-ESM 2010: model description and basic results of CMIP5-20c3m experiments, Geosci. Model Dev.*, **4**, 845–872, https://doi.org/10.5194/gmd-4-845-2011 (2011)

Zhang, S., G. Li, L. Tian, Q. Guo, X. Pan, Short-term exposure to air pollution and morbidity of COPD and asthma in East Asian area: A systematic review and meta-analysis. *Environ. Res.*, **148**, 15-23 (2016)

Zheng, X. Y., H. Ding, L. N. Jiang, S. W. Chen, J. P. Zheng, M. Qiu, Y. X. Zhou, Q. Chen, W. J. Guan, Association between Air Pollutants and Asthma Emergency Room Visits and Hospital Admissions in Time Series Studies: A Systematic Review and Meta-Analysis. *PLoS One*, **10**, e0138146 (2015)

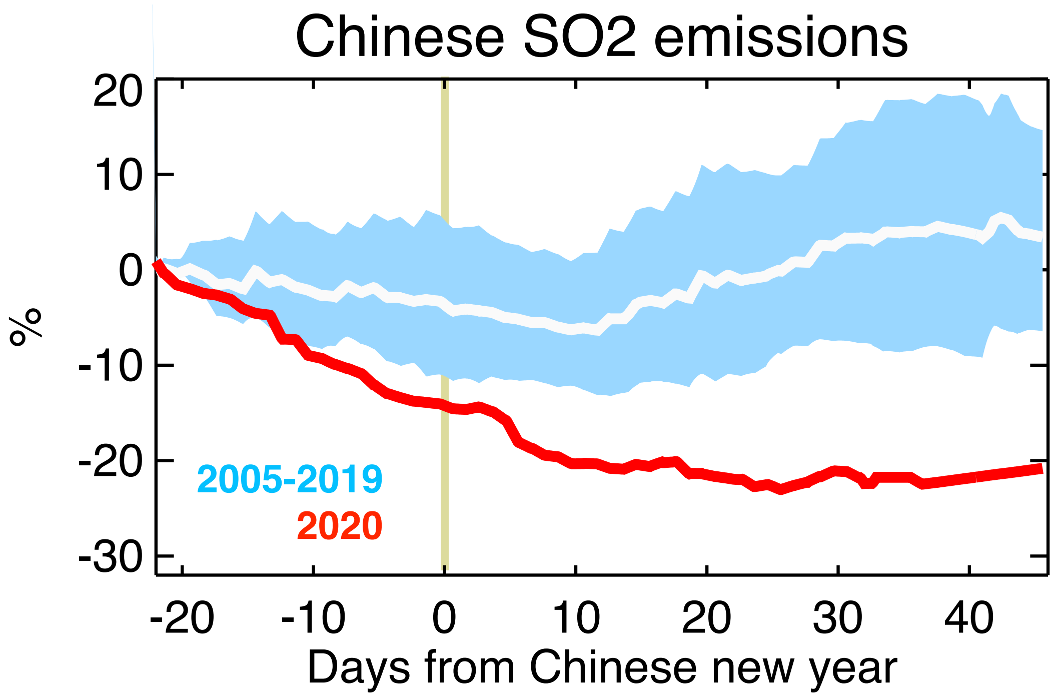

Fig S1. Time series of relative changes in Chinese SO_2 emissions (in %) as a function of days from CNY for 2005-2019 (mean by while line and 1-σ standard deviation in light blue shade) and 2020 (red line).

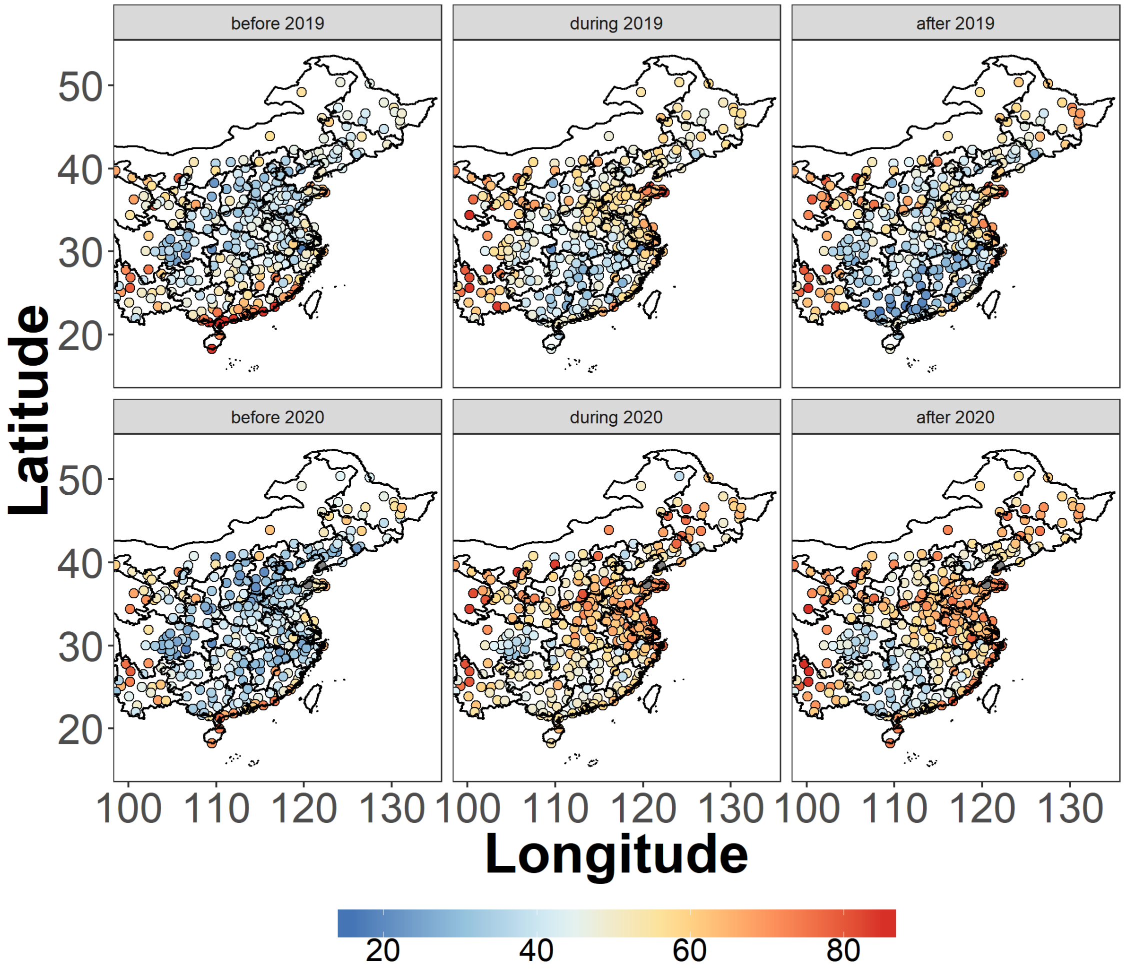

Fig S2. Spatial distributions of surface ozone concentrations (in $\mu g m^{-3}$) from the NAQMS stations averaged over two weeks before (left), during (center), and two weeks after (right) the Chinese new year holiday in 2019 (top) and 2020 (bottom).

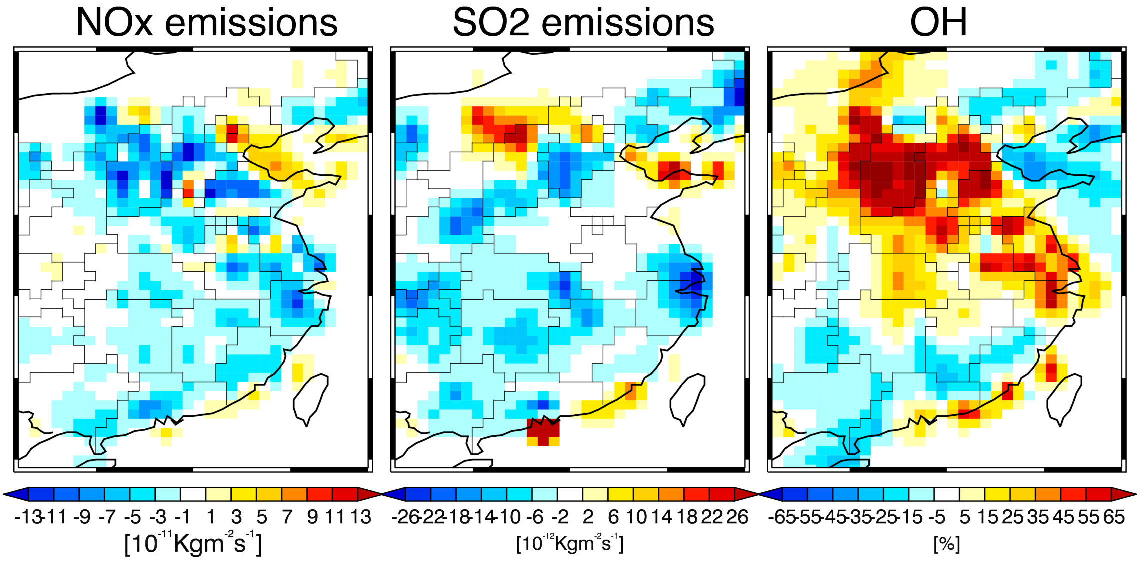

Fig S3. Differences in surface NOx emissions (left) and surface SO_2 emissions (center) between the standard and adjusted emissions averaged over February 15-25, 2020. The adjusted emissions were produced based upon the difference between 2015-2019 emission trends after January 23 and those in 2020 to follow the average recovery in 2015-2019. The relative changes in OH concentrations (in %) obtained from the model sensitivity simulations using the two emission data are also shown (right).

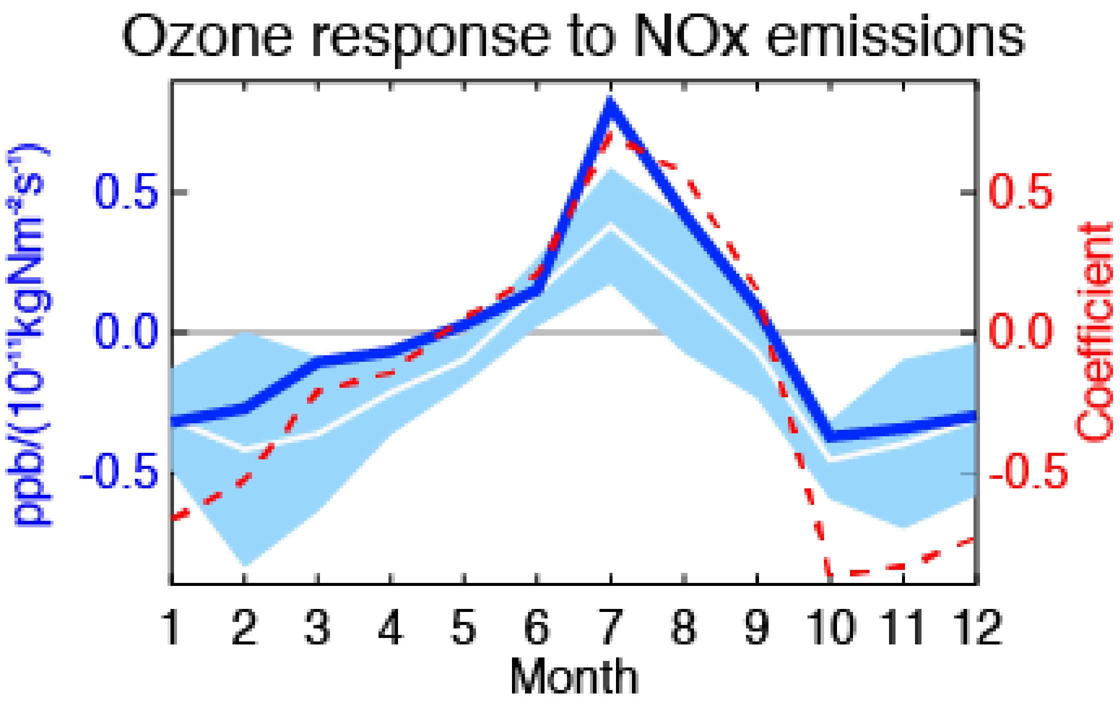

Fig S4. Time series of model response of surface ozone concentrations to NOx emissions estimated from linear regressions using the multi-model integrated fields in 2007 for northeastern China (110–121°E, 32–42°N). The 1σ deviation among the four models (i.e., model spread) is shown in light blue. The multi-model mean value (i.e., an average of individual estimates) is shown by white lines. The correlation is shown by dashed lines.

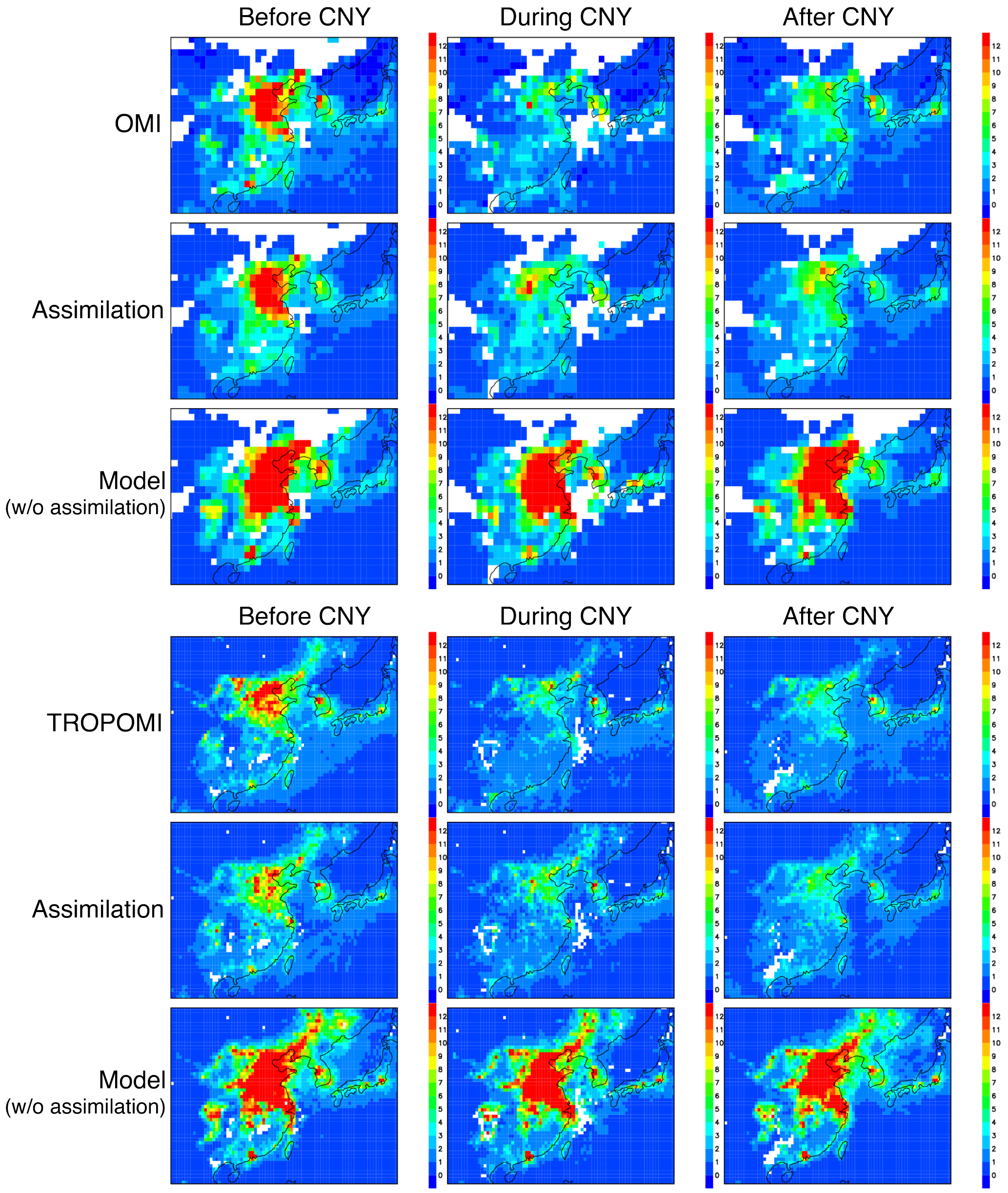

Fig S5. Spatial distributions of tropospheric NO_2 columns (in $10^{15} molec\, cm^{-2}$)averaged over two weeks before (left), during (center), and two weeks after (right) the CNY holiday. Upper row shows the tropospheric NO_2 columns obtained from the assimilated satellite retrievals, center row shows the data assimilation results; and lower row shows the data assimilation results. The results are shown for OMI (upper panels) and TROPOMI (lower panels) assimilation results.

Time	NO_2		O_3		PM2.5	
	$\mu g\, m^{-3}$	Δ[%]	ppb	Δ[%]	$\mu g\, m^{-3}$	Δ[%]
2019 bef	33.0±0.7		22.0±0.7		61.1±0.7	
2019 dur	15.7±0.4	-52.3	24.1±0.7	9.8	50.3±0.6	-17.7
2019 aft	28.4±0.7	80.8	22.0±0.9	-8.6	55.2±0.8	9.8
2020 bef	34.1±0.7		19.1±0.9		67.7±0.7	
2020 dur	16.7±0.5	-50.9	27.1±1.0	41.5	60.2±0.6	-11.1
2020 aft	16.6±0.4	-1.0	26.7±0.5	-1.4	45.8±0.7	-23.9

Table S1. The observed concentrations of NO_2 (in $\mu g\, m^{-3}$), O_3 (in ppb), and PM2.5 (in $\mu g\, m^{-3}$) from the national air quality monitoring stations (NAQMS) stations averaged during two weeks before, during, and two weeks after the CNY holidays in 2019 and 2020. The relative changes (Δ[%]) between before and during and between during and after the CNY holiday are also shown. The estimated random and sampling errors are shown as the measurement uncertainty.

		China				NE China				SE China			
Instrument	Time	NOx		SO_2		NOx		SO_2		NOx		SO_2	
		E	Δ[%]	E	Δ[%]	E	Δ[%]	E	Δ[%]	E	Δ[%]	E	Δ[%]
OMI	Bef	7.6		5.3		2.5		1.4		3.2		1.4	
	Dur	6.7	-12.2	5.0	-6.9	2.0	-21.8	1.3	-7.4	2.8	-11.5	1.3	-12.5
	Aft	6.2	-7.2	4.6	-7.1	1.9	-2.0	1.2	-4.0	2.4	-14.8	1.3	0.0
	ΔTotal		-18.5		-13.3		-23.4		-11.1		-24.6		-12.5
TROPOMI	Bef	5.1				1.3				2.0			
	Dur	4.3	-14.1			1.1	-18.8			1.7	-16.3		
	Aft	4.2	-4.4			1.0	-11.1			1.5	-11.2		
	ΔTotal		-17.8				-27.8				-25.7		

Table S2. Total regional emissions (E) of NOx (TgN/yr) and SO_2 (TgS/yr) for China, northeastern China (110–123°E, 35–45°N), and southeastern China (110–123°E, 22–35°N) obtained from the data assimilation results averaged over two weeks before, during and two weeks after the CNY holidays in 2020. The relative changes (Δ[%]) between before and during and between during and after the CNY holiday are also shown. ΔTotal represents the relative changes between before and after the CNY holiday.

Data	NE China	SE China
NOx emission (OMI)	-34.7	-34.5
NOx emission (TROPOMI)	-35.0	-37.1
NO_2 column (OMI)	-33.9	-42.0
NO_2 column (TROPOMI)	-50.6	-38.2

Table S3. Relative changes in surface NOx emissions and tropospheric NO_2 columns (in %) derived from OMI and TROPOMI measurements separately from January 4-14, 2020 to February 14-24, 2020. The results are shown for northeastern (NE) China (110–122°E, 35–42°N) and southeastern (SE) China (110–122°E, 22–35°N).

	NO_2			PM2.5			MDA8 O_3		
	Δ(o)	Δ(m)	Bias	Δ(o)	Δ(m)	Bias	Δ(o)	Δ(m)	Bias
Liaoning	-19.5	-21.7	1.4±1.2	-14.4	2.2	-48.6±29.7	5.0	4.4	-2.2±2.5
Beijing	-11.7	-14.9	22.2±9.4	25.9	-12.2	-14.5±34.5	5.8	2.5	2.3±2.1
Tianjin	-26.8	-10.5	23.3±11.9	-37.5	-1.0	-13.2±23.7	8.7	4.3	1.4±2.8
Hebei	-29.1	-26.9	20.5±4.9	-34.4	-14.0	-15.7±12.7	10.7	5.8	-1.5±2.8
Shanxi	-25.9	-37.6	19.3±6.6	-37.2	-20.8	-19.2±8.3	11.9	8.1	0.3±2.2
Shandong	-23.1	-31.7	15.3±4.1	-34.8	-3.0	4.2±13.7	6.9	10.7	-2.0±3.0
Jiangsu	-20.7	-21.4	22.2±4.2	-48.2	-22.7	8.6±10.5	8.7	12.1	2.2±4.5
Shanghai	-23.6	-25.6	20.0±4.6	-39.9	-5.1	4.8±14.2	9.8	11.0	6.4±4.9
Anhui	-18.7	-21.5	8.6±1.8	-44.2	-17.2	22.1±11.1	8.8	12.3	6.8±3.7
Henan	-25.8	-22.5	14.7±6.1	-59.2	-29.8	-10.5±13.3	13.1	12.3	-2.0±2.7
Hubei	-15.3	-6.1	3.1±5.6	-22.4	-21.4	33.5±7.7	8.3	12.3	8.9±3.2
Zhejiang	-19.9	-15.8	12.4±2.0	-14.8	-4.4	21.4±5.3	10.4	2.9	10.1±5.7
Jiangxi	-13.8	-7.8	3.8±5.1	-11.6	-11.0	26.0±6.0	8.0	1.3	10.3±5.5
Hunan	-10.5	-3.0	1.4±5.0	-14.4	-21.4	34.1±6.5	1.9	1.9	15.9±4.2
Guizhou	-7.8	0.7	1.2±4.0	-0.5	6.7	33.6±5.5	3.3	-2.1	19.7±2.7
Fujian	-10.3	-10.6	4.6±3.7	-6.0	-8.7	8.3±6.9	2.9	-0.5	8.5±2.4
Guangdong	-12.4	-7.8	6.1±4.3	-4.5	-4.3	13.5±3.6	-4.9	-1.9	12.9±1.6
Guangxi	-7.1	-0.8	-3.7±3.9	-0.1	-4.1	14.7±1.8	-1.6	-4.6	18.8±5.1

Table S4. Comparisons of absolute changes in concentrations of MDA8 (ppb), PM2.5 ($\mu g\, m^{-3}$) and NO_2 ($\mu g\, m^{-3}$) between before and after the CNY holiday in 2020 from in-situ observations (Δ(o)) and model (Δ(m)). The mean model bias relative to the observation averaged between two weeks before and two weeks after the CNY holiday and its standard deviation across the three-time period (before, during, and after the CNY holiday) are also shown.

	Ozone			PM2.5					
	Asthma	Resp.	Total	Resp.	Card.	Card.(China)	Ch.bro.	Ch.asth.	Total
Liaoning	47	1	48	-24	-22	[-6]	-26	-433	-504
	(19–75)	(0–3)	(19–78)	(-60–12)	(-49–6)	(-49–1)	(-55–-1)	(-710–-189)	(-875–-173)
Beijing	87	3	89	-20	-18	[-5]	-25	-376	-439
	(70–104)	(2–3)	(71–107)	(-53–13)	(-43–7)	(-43–2)	(-56–6)	(-628–-124)	(-780–-97)
Tianjin	71	2	73	-0	-0	[-0]	-1	-11	-13
	(58–83)	(1–3)	(60–86)	(-32–31)	(-24–23)	(-24–5)	(-39–33)	(-261–235)	(-356–322)
Hebei	464	13	477	-198	-173	[-49]	-306	-3937	-4614
	(405–522)	(10–17)	(415–539)	(-333–-64)	(-272–-74)	(-272–-15)	(-544–-207)	(-5328–-3164)	(-6479–-3509)
Shanxi	220	6	226	-118	-105	[-30]	-149	-2235	-2607
	(188–251)	(5–8)	(193–260)	(-171–-66)	(-144–-66)	(-144–-14)	(-175–-74)	(-2497–-1696)	(-2986–-1901)
Shaanxi	27	1	27	-24	-22	[-6]	-28	-452	-527
	(-4–57)	(-1–3)	(-5–60)	(-76–27)	(-60–17)	(-60–4)	(-78–18)	(-851–-73)	(-1065–-12)
Shandong	571	17	588	-226	-196	[-56]	-361	-4530	-5313
	(495–678)	(12–21)	(507–669)	(-406–-47)	(-328–-64)	(-328–-13)	(-439–14)	(-5256–-2365)	(-6430–-2464)
Jiangsu	-183	-5	-188	-128	-115	[-33]	-141	-2340	-2723
	(-224–-141)	(-8–-3)	(-232–-144)	(-178–-78)	(-153–-77)	(-153–-16)	(-207–-126)	(-2855–-2120)	(-3394–-2403)
Shanghai	105	3	109	-20	-18	[-5]	-26	-384	-448
	(92–119)	(2–4)	(95–123)	(-45–4)	(-36–1)	(-36–0)	(-40–8)	(-509–-132)	(-631–-120)
Anhui	221	6	228	-213	-187	[-53]	-296	-4115	-4811
	(172–270)	(4–9)	(176–280)	(-322–-104)	(-268–-106)	(-268–-22)	(-476–-223)	(-5209–-3481)	(-6274–-3913)
Henan	440	13	453	-372	-324	[-92]	-579	-7401	-8675
	(347–532)	(8–18)	(355–550)	(-566–-178)	(-466–-180)	(-466–-38)	(-644–-150)	(-8093–-4956)	(-9769–-5465)
Hubei	85	3	88	-217	-189	[-54]	-317	-4244	-4967
	(37–133)	(0–5)	(37–138)	(-330–-104)	(-273–-106)	(-273–-22)	(-468–-199)	(-5227–-3428)	(-6298–-3837)
Zhejiang	339	10	349	-178	-160	[-46]	-196	-3254	-3788
	(290–388)	(7–13)	(297–400)	(-237–-120)	(-205–-115)	(-205–-24)	(139–236)	(2650–3519)	(2348–3520)
Jiangxi	-183	-5	-188	-128	-115	[-33]	-141	-2340	-2723
	(-224–-141)	(-8–-3)	(-232–-144)	(-178–-78)	(-153–-77)	(-153–-16)	(-207–-126)	(-2855–-2120)	(-3394–-2403)
Hunan	-278	-8	-285	-265	-234	[-67]	-352	-5072	-5923
	(-329–-226)	(-10.9–-5)	(-340–-231)	(-375–-155)	(-316–-152)	(-316–-32)	(-323–-93)	(-5124–-3416)	(-6989–-4668)
Guizhou	-174	-5	-179	-42	-37	[-11]	-63	-830	-972
	(-201–-146)	(-7–-3)	(-208–-150)	(-106–21)	(-84–10)	(-84–2)	(-147–24)	(-1329–-324)	(-1808–-402)
Fujian	-100	-3	-103	-41	-37	[-11]	-37	-712	-827
	(-135–-66)	(-5–-1)	(-139–-67)	(-62–-20)	(-54–-21)	(-54–-4)	(-47–-19)	(-818–-522)	(-1095–-697)
Guangdong	-146	-4	-151	-139	-126	[-36]	-146	-2514	-2925
	(-227–-66)	(-9–0)	(-235–-66)	(-220–-59)	(-187–-64)	(-187–-13)	(-149–-28)	(-2732–-1564)	(-3699–-2125)
Guangxi	-373	-11	-384	-81	-73	[-21]	-84	-1461	-1699
	(-407–-340)	(-13–-9)	(-419–-349)	(-124–-39)	(-106–-41)	(-106–-9)	(-101–-38)	(-1699–-1082)	(-2272–-1442)
Country	2046	60	2105	-2699	-2378	[-679]	-3707	-51907	-60,697
	(909–3182)	(-4–124)	(905–3306)	(-4745–-654)	(-3895–-861)	(-3895–-179)	(-6697–-716)	(-68166–-35648)	(-83503–-37879)

Table S5. The changes in ozone- and PM2.5-related morbidity due to the COVID-19 mitigation during February 15-25, 2020 based on the model sensitivity calculations with the standard and modified emissions. For ozone, total asthma-related ERVs for all age population and respiratory HAs in post-65 population were estimated. For PM2.5, the number of respiratory and cardiovascular HAs for all age population and cases of bronchitis in children ages 6-12 and asthma symptom days in children ages 5-19 were estimated. The results are shown for selected provinces and country total. The 95% upper and lower confidence levels are also shown in parentheses. The results for PM2.5

cardiovascular HAs using the China-specific estimates (Card. (China)) are also shown in square bracket.

	Ozone HA			PM2.5 HA		
	Changes	E(exposure)	E(conc)	Changes	E(exposure)	E(conc)
Liaoning	1	±2.0	-0.0±0.9	-46	±63.2	-0.3±23.5
Beijing	3	±1.0	0.0±0.2	-38	±58.3	0.0±17.3
Tianjing	2	±0.7	0.0±0.2	-1	±54.7	0.0±18
Hebei	13	±3.3	0.0±1.4	-371	±233.8	-4.5±63.7
Shanxi	6	±1.8	0.0±0.8	-223	±91.7	2.2±18.2
Shaanxi	1	±1.7	0.0±0.8	-46	±89.8	-0.2±20.8
Shandong	17	±4.3	0.0±2.2	-423	±311.2	10.7±62.8
Jiangsu	-5	±2.3	0.0±1	-243	±87.7	-2.2±15.1
Shanghai	3	±0.8	0.0±0.4	-38	±42.8	1.3±8.1
Anhui	6	±2.8	0.0±1.4	-400	±189.7	-1.7±36.8
Henan	13	±5.2	0.0±2.2	-696	±336.8	13.1±61.2
Hubei	3	±2.7	0.0±1.4	-406	±196.5	-1.2±32.5
Zhejiang	10	±2.8	0.1±0.1	-338	±103.3	32.8±1.6
Jiangxi	-5	±2.3	0.0±1	-243	±87.7	-2.2±15.1
Hunan	-8	±2.9	-0.1±1.4	-500	±192.0	12.6±20.5
Guizhou	-5	±1.6	-0.1±0.8	-79	±110.7	0.2±18.8
Fujian	-3	±1.9	-0.2±0.5	-78	±37	0.9±7.0
Guangdong	-4	±4.5	-0.0±2.4	-265	±142	5.3±31.3
Guangxi	-11	±1.9	-0.1±1	-155	±74.9	1.3±23.7
Country total	-60	±64	-1±28	-5077	±3563	428±826

Table S6. Total changes of respiratory HAs for short-term ozone exposure in post-65 population (Ozone HA), respiratory and cardiovascular HAs for short-term PM2.5 exposure (PM2.5 HA), and their uncertainty due to exposure-response functions (E(exposure), ±2-σ) and model concentration bias and its uncertainty (E(conc), bias±2-σ).

Data Set S1. Daily Chinese NOx emissions derived using OMI NO_2 (file name:"Miyazaki-ds01.nc").

Data Set S2. Daily Chinese NOx emissions derived using TROPOMI NO_2 (file name:"Miyazaki-ds02.nc").

Data Set S3. Daily Chinese SO_2 emissions derived using OMI SO_2 (file name:"Miyazaki-ds03.nc").

The data used in this paper are available for download at `https://ebcrpa.jamstec.go.jp/~miyazaki/data_GRL2020/`
